# Current Status of Gene Engineering Cell Therapeutics

**DOI:** 10.3389/fimmu.2018.00153

**Published:** 2018-02-05

**Authors:** Aurore Saudemont, Laurent Jespers, Timothy Clay

**Affiliations:** ^1^GlaxoSmithKline, Stevenage, United Kingdom; ^2^GlaxoSmithKline, Collegeville, PA, United States

**Keywords:** cell therapy, gene therapy, autologous, allogeneic, *ex vivo* therapy

## Abstract

*Ex vivo* manipulations of autologous patient’s cells or gene-engineered cell therapeutics have allowed the development of cell and gene therapy approaches to treat otherwise incurable diseases. These modalities of personalized medicine have already shown great promises including product commercialization for some rare diseases. The transfer of a chimeric antigen receptor or T cell receptor genes into autologous T cells has led to very promising outcomes for some cancers, and particularly for hematological malignancies. In addition, gene-engineered cell therapeutics are also being explored to induce tolerance and regulate inflammation. Here, we review the latest gene-engineered cell therapeutic approaches being currently explored to induce an efficient immune response against cancer cells or viruses by engineering T cells, natural killer cells, gamma delta T cells, or cytokine-induced killer cells and to modulate inflammation using regulatory T cells.

## Introduction

Cell and gene therapy is an emerging field with the high potential to offer a curative therapy. Gene therapy is defined as the use of genetic material such as DNA to manipulate a patient’s cells, and cell therapy is defined as the administration of live whole cells or of a specific cell population to a patient. In many diseases, cell and gene therapies are combined as gene engineering cell therapeutics in the development of promising therapies for the treatment of an acquired or inherited disease. The number of applications for gene engineering cell therapeutics is increasing at a very rapid pace, with these applications being at different development stages from preclinical to clinical.

Autologous gene engineering cell therapeutics have the potential to correct the underlying genetic cause of some monogenic disorders and potentiate immune responses against cancers to provide sustained clinical responses (1–5). In addition, one of the main advantages of autologous therapies is their full major histocompatibility complex (MHC) compatibility leading to a better engraftment and persistence of the cells and a low risk of graft versus host disease (GvHD). Gene transfer into autologous hematopoietic stem cells (HSC) has shown potential especially in treating primary immunodeficiencies such as X-linked severe combined immunodeficiency (X-SCID) or adenosine deaminase deficiency–SCID. The transfer of a chimeric antigen receptor (CAR) or T cell receptor (TCR) genes into autologous T cells allows redirecting the genetically engineered T cells towards specific antigens expressed on cancer cells or presented as peptides on MHC molecules, respectively. In particular, the transfer of autologous CD19-CAR T cells in patients with hematological malignancies has been very successful, achieving impressive remission rates (6). Notably, the Food and Drug Administration (FDA) recently approved the first CAR T cell therapy, Kymriah^®^ (or tisagenlecleucel), for patients with B cell acute lymphoblastic leukemia (ALL). In addition, another CAR therapy was approved by the FDA, “Yescarta^®^” (axicabtagene ciloleucel), for the treatment of adult patients with certain types of non-Hodgkin lymphoma.

**Figure 1:**
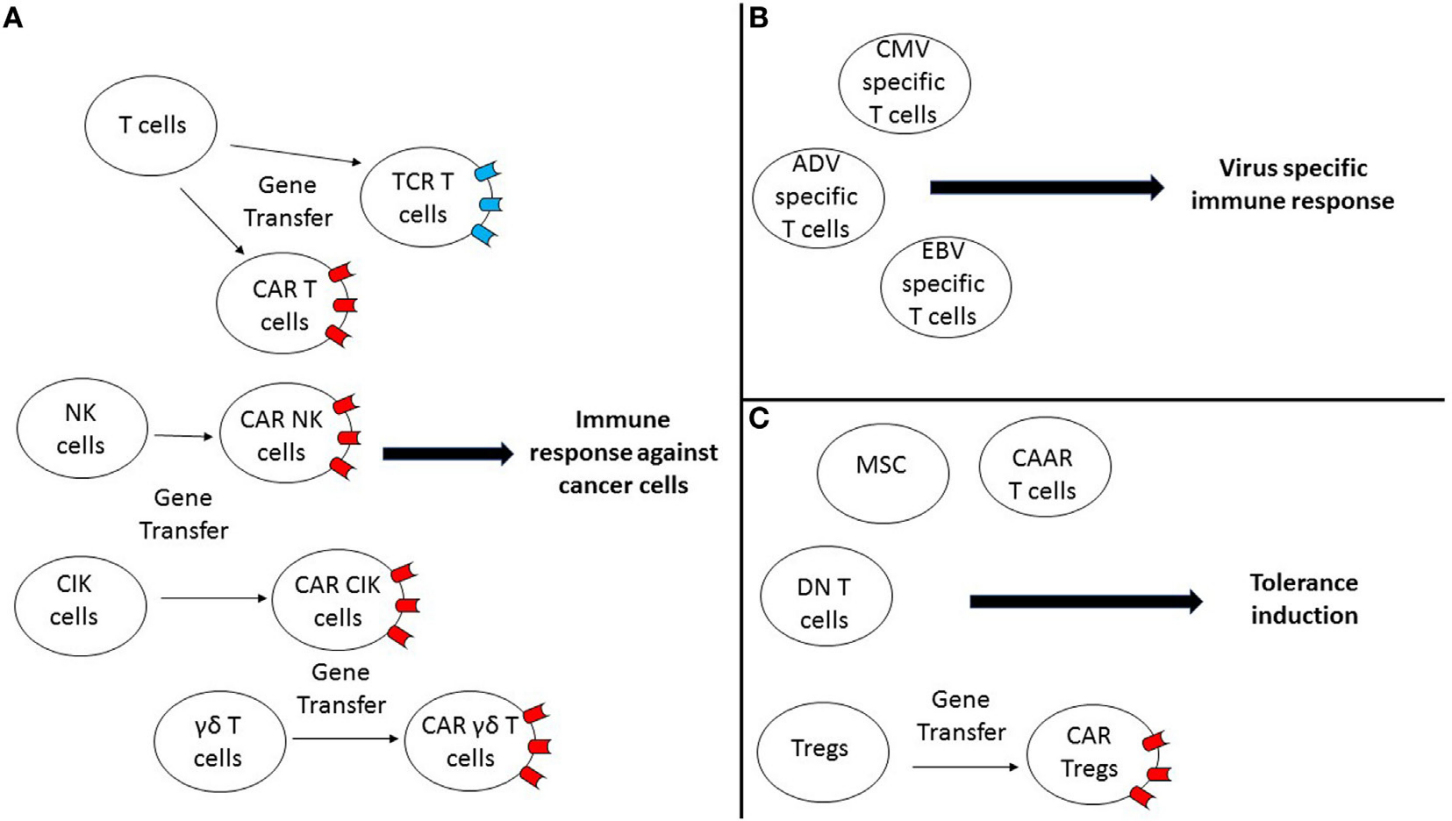
Gene-engineered cell therapeutic approaches are currently explored preclinically and clinically to induce potent immunity against cancer, infection, or to induce tolerance. **(A)** Different gene-engineered cell therapeutic approaches using either T cells, natural killer (NK) cells, cytokine-induced killer (CIK) cells, or γδ T cells are being explored to induce an efficient immune response against cancer cells. Notably, these different cell types can be reprogrammed by gene transfer of a T cell receptor (TCR) or a chimeric antigen receptor (CAR), so they can target efficiently specific antigens expressed by cancer cells. **(B)** Virus-specific T cells can be used as a cell therapy approach to restore virus-specific immunity in patients. **(C)** Different approaches are being explored to induce tolerance for different indications by either using mesenchymal stem cells (MSC), double negative (DN) T cells, CAR T cells, or regulatory T cells (Tregs)-based approaches or by explored manipulating Tregs (CAR-Tregs).

However, some of the trials testing gene engineering cell therapeutics have not been without setbacks such as the incidence of insertional mutagenesis observed in the first clinical trials for X-SCID, which has led to the design of new vectors allowing reducing their potential for insertional mutagenesis. This also highlighted the clear need for long-term follow-up for the patients receiving these live gene engineering cell therapeutics. In addition, several deaths linked to neurotoxicity in patients treated with CD19-CAR T cells have been reported and the cytokines produced after infusion of the product can lead to adverse effects such as cytokine release syndrome (CRS) that many patients experienced, highlighting the fact that we still need to gain a better understanding of the effects of gene engineering cell therapeutics in patients so as to make these therapies safer. Here, we review the latest gene engineered cell therapeutic approaches being currently explored preclinically but emphasizing those that have been clinically tested (Figure 1), to induce an efficient immune response against cancer cells or viruses by engineering T cells, natural killer (NK) cells, gamma delta T cells or cytokine-induced killer (CIK) cells and to modulate inflammation by using regulatory T cells (Tregs).

## Cell and Gene Therapies for Oncology

Different cell types can be used to develop gene engineering cell therapeutics to induce an immune response against tumor cells. Here, we will review the use of TCR- or CAR-modified T cells (TCR-T or CAR-T), NK cells, CIK cells, and gamma delta T cells for oncology.

### Autologous TCR-T and CAR-T

T cell engineering provides the possibility to generate antigen-specific T cells for many types of cancer. The identification of a graft versus leukemia (GvL) effect and subsequent recognition that T cells played a role provided an early indicator of the potential of T cells to mediate antitumor effects and led to the use of donor lymphocyte infusion (DLI). Subsequent studies of autologous tumor infiltrating lymphocytes (TIL) in melanoma patients for whom suitable TIL could be isolated and expanded successfully, allowing adoptive transfer, led to significant clinical responses (7, 8). The difficulty to isolate TIL from some patients’ tumors, especially with non-melanoma tumors, led to the concept of modifying autologous peripheral blood T cells to redirect them to recognize tumors offering an alternative approach to develop T cell therapies. Two principal strategies were developed, one using natural TCR that recognized human leukocyte antigen (HLA)-restricted tumor-associated antigen peptides (9–12) and the other seeking to use antibody recognition domains linked to TCR signaling molecules in CAR (13–15).

Both strategies have been continually refined over the last 15 years, with promising early data culminating in a number of medicines in late-stage clinical development and two approved medicines (Tables 1 and 2). The first clinical study of TCR-T was performed by Morgan et al. using a wild-type human TCR specific for a MART-1 epitope presented by HLA-A*0201, and the feasibility of this approach to modify autologous T cells *ex vivo* was demonstrated (12). There were some initial signs of clinical activity with 2/17 melanoma patients undergoing a partial response, and no related toxicities were observed. Subsequent studies included using TCR of mouse origin (16–20) and affinity-enhanced TCR (16, 21–23). A variety of different tumor antigen targets have also been evaluated in different tumor types (Table 1). The most advanced TCR-T clinical asset, NY-ESO-1-specific TCR-T cells, has shown particularly promising clinical responses in multiple myeloma (MM) and synovial sarcoma (19, 23, 24), with a 61% response rate and a 38% overall survival (OS) at 3 years (19, 24, 25) and a 91% response rate and a median OS of 3 years in MM in the context of autologous hematopoietic stem cell transplantation (HSCT) (23, 26). As shown in Table 1, improved clinical response rates have been observed in multiple TCR-T studies compared to the original study of Morgan et al. including complete responses, but there have also been some serious, sometimes fatal adverse events.

**Table 1:** Summary of key published clinical results of T cell receptor gene-modified T cell therapies.

Reference	Center	Clinicaltrials.gov identifier	Product	Indication	Clinical outcome	Toxicity
(12)	NCI	Phase I, approved by the NIH IRB, NCI IRB, NIH RAC, and FDA-CBER	Autol. T cells with MART-1-specfic wild-type hTCR	Melanoma	PR 2/17	No related toxicities
(16)	NCI	Phase I, NCI-07-C-0174 and NCI-07-C-0175	Autol. PB T cells with MART-1-specific high-affinity hTCR	Melanoma	PR 6/20	Up to G2 skin, G2 eye, and G3 ear toxicity
(16)	NCI	Phase I, NCI-07-C-0174 and NCI-07-C-0175	Autol. T cells with gp100-specific mTCR	Melanoma	CR 1/16, PR 2/16	Up to G2 skin, G2 eye, and G3 ear
(17)	NCI	Phase I, approved by the NIH IRB, NCI IRB, NIH RAC, and FDA-CBER	Autol. T cells with p53-specific mTCR	Various epithelial cancers	PR 1/14	
(18)	NCI	Phase I, NCT00923806	Autol. T cells with CEA-specific mTCR	Colorectal cancer	PR 1/3, 3/3 with decreased serum CEA protein levels	3/3 developed transient inflammatory colitis up to G3
(19)	NCI	Phase I, approved by the NIH IRB, NCI IRB, NIH RAC, and FDA-CBER	Autol. T cells with NYESO1-specific mTCR	Melanoma and synovial sarcoma	Melanoma CR 2/11, PR 3/11; synovial sarcoma PR 4/6	No related toxicity
(20)	NCI	Phase I, NCT01273181	Autol. T cells with MAGEA3-specific mTCR	Melanoma, synovial sarcoma, and esophageal cancer	Melanoma CR1/7, PR 4/7; synovial carcinoma 0/1; and esophageal carcinoma NR 0/1	3 patients developed mental disturbances and 2 died from necrotizing leukoencephalopathy
(21)	UPenn	Phase I, NCT01350401 and NCT01352286	Autol. T cells with affinity-enhanced NYESO1-specific hTCR	Melanoma and myeloma		2/2 patients died from cardiogenic shock
(22),	UCLA	Phase I, NCT00910650	Autol. T cells with affinity-enhanced MART1-specific hTCR	Melanoma	Short-term regression 9/14	2 patients experienced respiratory distress
(27)	MieU	Phase I, UMIN Clinical Trials Registry ID: UMIN000002395	Autol. T cells with MAGEA4-specific hTCR	Esophageal cancer	3/10 patients who had minimal tumor lesions at baseline survived for >27 months	No related toxicities
(23)	UPenn	Phase I, NCT01352286	Autol. T cells with affinity-enhanced NYESO1-specific hTCR	Multiple myeloma	nCR or CR 14/20, vgPR 2/20, PR 2/20, SD 1/20, and PD 1/20	Grade 3 or lower AE’s included 3/20 with Gr 3 GI aGVHD, 2/20 with Gr2 skin aGVHD

*aGVVD, acute graft versus host disease; Autol, autologous; CR, complete response; G2, grade 2; G3, grade 3; hTCR, human T cell receptor; MieU, Mie University; mTCR, murine T cell receptor; NCI, National Cancer Institute; nCR, near complete response (defined as myeloma monoclonal band detectable only by sensitive immunofixation assay); NR, no response; PD, progressive disease; PB, peripheral blood; Periph, peripheral; PR, partial response; SD, stable disease; UCLA, University of California Los Angeles; UPenn, University of Pennsylvania; vgPR, very good partial response (defined as ≥90% reduction in paraprotein levels)*.

Early CAR-T studies, using what is now termed a first-generation construct design with the antigen-binding domain scFV linked to CD3 zeta or FcR γ chain as a signaling domain, provided evidence of feasibility for CAR-T cell production but lacked clinical efficacy in HIV-infected patients or patients with solid tumors (Table 2) (28–30, 32, 33). Indeed, activation through the CD3zeta chain or FcR γ chain was insufficient to produce productive immunity (43, 44). Second-generation construct designs were developed by addition of a costimulatory domain (typically CD28 or 41BB) to CD3zeta to simultaneously provide both activation and co-stimulatory signals [several recent reviews have addressed CAR design in detail (45–49)]. The CAR-T field was truly energized when Carl June and colleagues at the University of Pennsylvania, using second generation CD19-specific CAR-T cells, achieved two complete responses in the treatment of three patients with refractory advanced chronic lymphocytic leukemia (CLL) using anti-CD19 CAR T cells (4, 35). Subsequently, CD19 CAR-T cell therapy has generated complete and durable remissions in patients with refractory and relapsed B cell malignancies (5, 6, 37, 50). As previously mentioned, the FDA has recently approved the CD19 CAR-T cell approach from Novartis, Kymriah^®^, to treat patients with B cell ALL and the CD19 CAR-T cell approach from Kite Pharma, YESCARTA™, for the treatment of diffuse large B-cell lymphoma.

**Table 2:** Summary of some key published clinical results of CAR-gene-modified T cell therapies.

Reference	Center	Clinicaltrials.gov identifier	Product	Indication	Outcome	Toxicity
(28–30)	Cell Genesys Inc.	Phase I, NCT01013415	Autol. CD4zeta-modified CAR T cells	HIV-infected subjects	Prolonged CAR T survival (detectable at >decade) and trafficking to infected tissues	
(30, 31),	Cell Genesys Inc.	Phase II, NCT01013415	Autol. CD4zeta-modified CAR T cells plus ART	HIV-infected subjects with undetectable plasma viremia	Prolonged CAR T survival (detectable at >decade) trend toward fewer patients with recurrent viremia	
(32)	NCI	Phase I NCI, approved by the NIH IRB, NCI IRB, NIH RAC, and FDA-CBER	Autol. CAR T cells specific for alpha-folate receptor in combination with high-dose IL-2 or allogeneic PBMC	Ovarian cancer	No reduction in tumor burden, short CAR T persistence	Mild side effects, Gr1 and 2 with Gr3 and Gr4 toxicities likely IL-2-related in patients receiving high-dose IL-2
(33)	EUMC	Phase I, approved by Dutch regulatory authorities	Autol. CAR T cells specific for carbonic anhydrase IX	Metastatic renal cell carcinoma	PD 3/3	3/3 developed Gr2-4 liver toxicity likely on target toxicity related to the CAR T cells, 3/3 developed HAMA to the CAR scFv observed
(34)	NCI	Phase I, NCT00924326	Autol. Murine scFv CAR T cells specific for CD19	Advanced follicular lymphoma patient case report	A durable PR lasting 32 weeks before progressing with CD19^+^ disease	
(4, 35)	UPenn	Phase I, NCT01029366	Autol. CAR T cells specific for CD19	Advanced, chemotherapy-resistant CLL	CR 2/3, PR 1/3. CAR T cells expanded up to 10,000 fold, trafficked to the BM, and persisted for >6 months in the peripheral blood	Gr3 tumor lysis syndrome (1/3) patients. 3/3 persistent B-cell aplasia
(36)	MSKCC	Phase I, NCT00466531 and NCT01044069	Autol. CAR T cells specific for CD19	Patients with chemotherapy-refractory CLL or relapsed B-cell ALL	CLL PR 1/8, SD 2/8, ALL B-cell aplasia 1/1	Well tolerated, most patients had rigors, chills, and transient fevers. 1 death from sepsis
(5)	MSKCC	NCT01044069	Autol. CAR T cells specific for CD19	Relapsed or refractory B cell ALL	CR 14/16 (88%)	Severe CRS 44%, CNS toxicity 38%
(37)	UPenn/CHOP	Phase I, NCT01626495 and NCT01029366	Autol. CAR T cells specific for CD19	Relapsed or refractory ALL (25 pediatric, 5 adults)	CR 27/30	Severe CRS 27%, CNS toxicity 43%
(35)	UPenn	Phase I, NCT01029366	Autol. CAR T cells specific for CD19	Relapsed or refractory CLL (14 adults)	CR 4/14, PR 4/14	GR 3 or 4 CRS 43%, CNS toxicity 36%
(38)	NCI	Phase I, NCT00924326	Autol. Anti-CD19 CAR T cells	Advanced B-cell malignancies (9 DLBCL, 2 indolent lymphomas, and 4 CLL)	CR 8/15 (DLBCL 4/7 evaluable patients), PR 4/15, SD 1/15, NE 2	CRS 1/15, CNS toxicity 25%
(39)	NCI	Phase I, NCT01593696	Autol. Anti-CD19 CAR T cells	Children and young adults (aged 1–30 years) with relapsed or refractory ALL (20) or NHL (1)	CR 14/20 ALL	Gr3 and 4 CRS 30%, CNS toxicity 30%
(40)	FHCRC	Phase I, NCT01865617	Autol. Anti-CD19 CAR T cells with defined CD4:CD8 composition	Adult B cell ALL after lympho-depleting chemotherapy	CR: 27/30, MRD 2/30, and NE 1/30	
(41)	MCC	Phase I, NCT02348216	Autol. Anti-CD19 CAR T cells	Refractory DLBCL	CR: 4/7	Gr4 CRS 1/7, CNS toxicity Gr4 1/7, and Gr3 2/7
(42)	BCM	Phase I, NCT01316146	Autol. Anti-CD30 CAR T cells	Relapsed/refractory HL or ALCL	HL CR 2/7, SD 2/7, and ALCL CR 1/2	No toxicities attributable to anti-CD30 CAR T

*ALL, acute lymphoblastic leukemia; ALCL, anaplastic large cell lymphoma; ART, anti-retroviral therapy; BCM, Baylor College of Medicine; CAR, chimeric antigen receptor; CHOP, Children’s Hospital of Philadelphia; CLL, chronic lymphocytic leukemia; CNS, central nervous system; CR, complete response; CRS, cytokine release syndrome; DLBCL, diffuse large B-cell lymphoma; EUMC, Erasmus University Medical Center; FHCRC, Fred Hutchinson Cancer Research Center; G3, grade 3; G4, grade 4; HAMA, human anti-mouse antibodies; HIV, human immunodeficiency virus; HL, Hodgkin lymphoma; IL-2, interleukin 2; MCC, Moffitt Cancer Center; MRD, minimal residual disease; MSKCC, Memorial Sloan Kettering Cancer Center; NCI, National Cancer Institute; NE, non-evaluable; NHL, non-Hodgkin lymphoma; PBMC, peripheral blood mononuclear cells; PD, progressive disease; PR, partial response; UPenn, University of Pennsylvania*.

These autologous CAR-T and TCR-T cell therapies show great promise but there remain many aspects of these approaches that need to be further refined. The highly potent nature of these modified T cells produces dramatic tumor regressions; however, toxicities have also been observed. There have been a number of deaths on TCR-T and CAR-T trials. A detailed review of toxicity is beyond the scope of this review, but it is important to briefly underscore the risks. Some of these deaths have been due to apparent on target, off tumor toxicity, where expression of the target antigen on normal tissues occurs. For example, a patient died from rapid respiratory failure and multiorgan dysfunction in a CAR-T trial targeting ErbB2 in patients suffering from lung carcinoma, due to the recognition of ErbB2 on normal lung cells (51). Both Morgan et al. and Linette et al. reported instances where TCR-T targeting different MAGEA3 epitopes led to reactivity against proteins with amino acid sequence homolog to the target epitope in the brain (MAGEA12) and the heart (Titin), respectively (20, 21) (Table 1). In various CD19 CAR-T trials, patient deaths have been reported due to neurotoxicity caused by cerebral edemas [reviewed in Ref. (52)]. Here, the exact mechanism is less clear. Other severe side effects, such as CRS and tumor lysis syndrome, can also mean that patients often require aggressive support in an intensive care unit setting (53).

Another area for improvement is to induce better efficacy in more indications. Indeed, clinical response rates and the durability of response in some hematologic malignancies and all solid tumors are currently lower than those seen with CD19 CAR-T in ALL. The tumor microenvironment in solid tumors is a hostile environment for T cells, and low persistence of gene-engineered T cells with low efficacy has been observed. Since there is a dearth of truly tumor-specific targets because most tumor-associated antigens are also naturally expressed on some normal tissues, there is a need for new targets and ways to better control “on target, off-tumor” toxicity. Additional strategies will likely be needed to improve the effectiveness of CAR-T and TCR-T in the face of these obstacles [reviewed in Ref. (49, 54)]. In addition, the scale-up in manufacturing capability to supply thousands of patients has yet to be demonstrated and will be critical for the success of these autologous T cell approaches.

### NK Cells

Natural killer cells are defined as CD56^+^CD3^−^ cells and are innate immune lymphocytes able to exert cytotoxicity against tumor cells and virally infected cells without prior stimulation. Because of their antitumor activity, NK cells have been put forward as potential candidates for cancer immunotherapy. Cell therapy using autologous NK cells has proven to be safe and feasible when treating hematological malignancies such as CLL or different types of solid tumors but with however quite limited clinical responses (55–59).

The first evidence that allogeneic NK cells could be more cytolytic against tumor cells than autologous NK cells stems from the study by Ruggeri et al., who showed that allogeneic NK cells were very potent effectors of the GvL especially in the case of a killer-cell immunoglobulin-like receptors ligand incompatibility in the graft versus host direction in patients who received HLA-mismatched HSCT (60). In addition, the infusion of allogeneic NK cells together with interleukin (IL)-2 has shown some promising results in patients with different types of advanced cancers (61, 62) and several groups reported similar results using allogeneic NK cells mainly in the context of haploidentical HSCT (63–69) (Table 3). Although NK cells are short-lived, they may provide a potential off-the-shelf therapy with limited toxicity as they do not induce GvHD. A recent first-time-in-human study has also been reported exploring the use of umbilical cord blood (CB)-derived NK cells in MM (70).

**Table 3:** Summary of some key published clinical results of NK cell therapies, including CAR-NK cells.

Reference	Center	Clinicaltrials.gov identifier	Product	Indication	Outcome	Toxicity
(60)	PUSM	Phase I, unknown	KIR-matched or KIR-mismatched allogeneic NK cells	AML, ALL	KIR-mismatch independently predicted survival in AML, confirming an NK-mediated GvL effect	
(61)	UMinn	Phase I, approved by the UMinn IRB and conducted under BB-IND 8847	Haploidentical, related-donor NK cell infusions	Metastatic melanoma, metastatic RCC, or poor-prognosis AML	Infusions after Hi-Cy/Flu conditioning led to increased endogenous IL-15, expansion of donor NK cells, and induction of complete hematologic remission in 5 of 19 poor-prognosis AML patients	
(71)	UAMS	Phase I, approved by the UAMS IRB and conducted under BB-IND 11347	Haploidentical, T-cell depleted, KIR ligand-mismatched NK cells, followed by delayed rescue with autologous stem cells	Advanced MM	CR 3/10, nCR 2/10, MR 1/10, PR 1/10, SD 1/10, and PD 2/10	Haploidentical NK cell infusions were safe and did not impair engraftment or cause GvHD
(66)	St. Judes	Phase I, NCT00187096	Haploidentical NK cells	AML	All patients had transient engraftment and expansion of NK cells *in vivo*	Well tolerated
(58)	NCI	Phase I, NCT00328861	Autologous NK cells	Metastatic melanoma or RCC	No clinical responses were observed; evidence of NK cell persistence but decreased expression of NKG2D, and lack of *ex vivo* cytotoxicity	
(72)	UMinn	Phase II, approved by the UMinn IRB and conducted under BB-IND 8847	Haploidentical related donor NK cells	Breast or Ovarian cancer	*In vivo* expansion of donor NK cells failed and host Tregs increased	
(67)	UB	Phase I, NCT00799799	Haploidentical KIR ligand-mismatched NK cells	AML	CR 1/5 with patients with active disease. CR 2/2 patients with a molecularly relapse	No NK cell–related toxicity, including GVHD
(73)	RUMC	Phase I, approved by the IRB and was performed under an FDA IND for the *ex vivo* expansion of NK-92 cells	Allogeneic NK92 cell line	Refractory metastatic RCC (*n* = 11); refractory metastatic melanoma (*n* = 1)	PD 10/12. Transient mixed response 1/12. Minor response 1/12	Infusional toxicities were generally mild, with one grade 3 fever and one grade 4 hypoglycemic episodes. All toxicities were transient and resolved
(74)	Multicenter trial	Phase I, study approved by the ethics committee at the University of Frankfurt/Germany, Germany	Allogeneic NK92 cell line	Treatment-resistant solid tumors/sarcomas (n = 13) or leukemia/lymphoma (n = 2)	MR 2/15, SD 1/15, and PD 12/15. The cells persisted in the recipient’s circulation for at least 48 h	No infusion-related or long-term side effects were observed. Infusions of NK-92 cells up to 10^10^ cells/m2 was well tolerated
(75)	PMCC	Phase I, NCT00990717	Allogeneic NK92 cell line	Lymphoma or multiple myeloma patients who relapsed after AHCT for relapsed/refractory disease (*n* = 12)	5/12 patients exhibited a response, 1/12 PR, 2/12 CR (one sustained CR with patient alive 10 years after therapy), 1/12 transient response, 1/12 mixed transient response	Acute infusion-related toxicity (gr1-2 fever and chills) (N = 4/12) that subsided with symptomatic management
(76)	UPMC	Phase I, NCT00900809	Allogeneic NK92 cell line	Refractory/relapsed AML (*n* = 7)	Transient activity seen in 3 of 6 evaluable patients	One patient developed grade 2 fever and chills following each aNK cell infusion that required hospitalization; these effects were reversible with supportive care
(62)	UAMS	Phase I, approved by the UAMS IRB and conducted under BB-IND 14560	Autologous or haploidentical-related donor NK cells expanded in culture with a K562-mb15-41BBL cell line	High-risk relapsed MM	PR 1/7, significant *in vivo* expansion associated with fresh cells	No related SAE
(70)	Phase I, MDACC	NCT01729091	CB NK cells	MM	10 patients achieved partial responses, including eight with a “near complete response”	No infusional toxicities and no GvHD
Study PI: David Shook	St. Judes	Phase I, NCT00995137	Haploidentical donor NK CAR-NK	B-ALL	Data not yet reported	
PI’s: Poh Lin Tan, Dario Campana	NUHS	Phase I, NCT01974479	Allogeneic donor CD19-CAR-NK	B-ALL	Data not yet reported	
PI: Katy Rezvani	MDACC	Phase I, NCT03056339	CB-derived CD19 CAR-engineered NK Cells	B Lymphoid malignancies	Data not yet reported	

*AHCT, autologous hematopoietic cell transplantation; ALL, acute lymphoblastic leukemia; AML, acute myeloid leukemia; CR, complete response; GvHD, graft versus host disease; GvL, graft versus leukemia; Hi-Cy/Flu, high-dose cyclophosphamide and fludarabine; KIR, killer-cell immunoglobulin-like receptor; MDACC, M.D. Anderson Cancer Center; MM, multiple myeloma; MR, mixed response; NCI, National Cancer Institute; NUHS, National University Health System, Singapore; nCR, near complete response; PD, progressive disease; PMCC, Princess Margaret Cancer Centre, Toronto; PUSM, Perugia University School of Medicine; PR, partial response; RCC, renal cell carcinoma; RUMC, Rush University Medical Center; SAE, serious adverse event; St. Judes, St. Judes Children’s Research Hospital; UAMS, University of Arkansas for Medical Sciences; UB, University of Bologna; UMinn, University of Minnesota; UPMC, University of Pittsburgh Medical Center*.

A number of gene engineering cell therapy using human NK cell lines have been established. In particular, NK92 has been investigated as a therapy, either unmodified or with a variety of different genetic modifications. The first human clinical trial report using NK92 cells by Arai et al. showed some initial signs of clinical activity (73). Subsequently, a multicenter trial enrolled 15 patients, showing as well signs of clinical activity (74). Recently, clinical trials results with NK92 cells were reported by Keating and colleagues at the Princess Margaret Cancer Centre, Toronto (NCT00990717) (75). In that study, 5 of 12 patients exhibited a clinical response, including 2 complete responses, 1 of which is sustained and ongoing 10 years posttherapy. Boyiadzis et al. also recently reported results of a clinical trial of NK92 cells in 7 refractory/relapsed AML patients with 3/6 showing transient clinical responses (76).

Since NK92 cells do not express CD16 and therefore cannot mediate ADCC, a NK92 variant that has been engineered to express CD16 and intracellularly retained IL-2 (haNK) to be combined with monoclonal antibody therapies and clinical development is underway (e.g., NCT03027128). No clinical results with haNK cells have yet been reported.

Natural killer cells could also be used for the generation of CAR NK cell products for immunotherapy to enhance their effector function by providing antigen specificity. This could be especially effective in the case of cancers resistant to NK cell killing. Moreover, CAR-NK cells could be used to bridge the patient’s immunity during HSCT or prior to administration of another therapy such as CAR-T cells. Preclinical studies using CAR-NK cells have shown promising results *in vitro* and in animal models against different types of solid tumors (77, 78) and hematological malignancies (79–81). Thus, first-time-in human testing is underway. Notably, Shook and colleagues at St. Judes Children’s Research Hospital, Memphis, USA, have completed enrollment in a trial of CD19 CAR-expressing NK cells for B-Lineage ALL (NCT00995137), and results are awaited. The CAR used was a second-generation design with a 4-1BB costimulatory domain linked to the CD3 zeta chain. Tan, Campana and colleagues at the National University Health System in Singapore are using the identical CAR construct in an actively recruiting trial of haploidentical CD19 CAR-expressing NK cells for B-Lineage ALL (NCT01974479). The number of early clinical trials of CAR-NK is increasing year-on-year. The Chinese company PersonGen BioTherapeutics has opened four studies to enrollment in 2016, using a third-generation CAR design with the relevant scFV attached to CD28 and 41BB costimulatory domains and the CD3 zeta chain in transduced allogeneic NK92 cells. The trials are focused on different targets and indications: CD7^+^ leukemia/lymphomas in adults (NCT02742727), CD33^+^ myeloid malignancies in children and adults (NCT02944162), refractory CD19^+^ malignancies in children and adults as bridge to HSCT transplant (NCT02892695), and MUC1^+^ solid tumors including malignant glioma of brain, colorectal carcinoma, gastric carcinoma, hepatocellular carcinoma (HCC), non-small-cell lung cancer (NSCLC), pancreatic carcinoma, and triple-negative basal-like breast carcinoma (NCT02839954). Early in 2017, Rezvani and colleagues at the MD Anderson Cancer Center, Houston, TX, USA and Bellicum Pharmaceuticals, Houston, TX, USA opened a trial of umbilical and CB-derived CAR-engineered NK cells for B lymphoid malignancies that is currently recruiting patients (NCT03056339).

Similarly, CAR-modified NK92 cells have been tested in preclinical studies and shown promising results (82–84). Consequently, several CAR-modified NK92 clinical studies are underway but currently no clinical results have been published. Overall, all the ongoing clinical trials will provide key data on safety and efficacy of gene engineering NK cell therapeutics. It will be interesting to evaluate whether NK cell therapies can be as effective as their T cell counterparts for certain indications and whether CAR-NK cells lead to similar adverse effects such as CRS and neurotoxicity.

### CIK Cells

Cytokine-induced killer cells are a heterogeneous effector CD3^+^ CD8^+^ cell population that exhibit non-MHC-restricted cytotoxicity [reviewed in Ref. (85)]. There has been a long history of clinical studies testing CIK cells, with evidence of antitumor effects of CIK cells against hematologic malignancies and solid tumors, along with studies exploring their antivirus potential and anti-GVHD potential. The first human clinical trial of autologous peripheral blood-derived CIK transfected to express human IL-2 by electroporation demonstrated the safety in patients with metastatic renal cell carcinoma, colorectal carcinoma, and lymphoma (86) (Table 4). In one patient with follicular lymphoma grade I, a pre-existing bone marrow involvement as the only sign of disease resolved after CIK cell therapy and was scored as a complete clinical response.

**Table 4:** Summary of some key published clinical results of CIK cell therapies.

Reference	Center	Clinicaltrials.gov identifier	Product	Indication	Outcome	Toxicity
(86)	HUB		Autologous peripheral blood CIK transfected to express IL-2	RCC, colorectal carcinoma, and lymphoma	CR 1/10, SD 3/10, and PD 6/10	3/10 patients developed gr2 fever that resolved the next day with or without the addition of antibiotics
(87)	TMUC	Phase II, approved by the State Food and Drug Administration of China (2006L01023) and by the ethics committee of Cancer Hospital of Tianjin Medical University	Autologous CIK plus/minus chemotherapy	NSCLC, *n* = 87 patients per treatment arm	Significantly higher 3-year OS rate and median OS time in CIK among early-stage patients and in advanced-stage patients, significantly improved 3-year PFS and OS rates in CIK group	Not reported
(88)	SUSM	Phase II, NCT00699816	Anti-CD3-activated CIK	HCC (*n* = 230)	RFS 44 months in the immunotherapy group and 30 months in the control group (*p* = 0.010)	Significantly more AE in the immunotherapy group (*p* = 0.002), but no significant difference in the proportion of patients with serious AEs between groups (*p* = 0.15)
(89)	GFCH	The study protocol received ethical approval from the Regional Ethics Committee of Guangzhou Fuda Cancer Hospital	Autologous CIK together with monocyte-derived autologous DC plus freeze-thawed tumor lysate	Breast cancer, immunotherapy treatment group, *N* = 188 patients and chemotherapy alone control group, *N* = 180 patients	DFS and OS were both significantly prolonged in patients in the DC-CIK treatment group compared to the control group (*p* < 0.01)	The most common AE was fever in 34.6% of patients. Information on AE grade was not reported
(90)	SUSM	Phase III, NCT 0807027	Autologous CIK plus chemoradiotherapy (*n* = 91) versus chemoradiotherapy alone (*n* = 89)	Newly diagnosed glioblastoma	Improved median PFS but no difference in OS between the CIK and control groups	Grade 3 or higher adverse events, health-related quality of life and performance status between the two groups did not show a significant difference

*B-ALL, B-acute lymphoblastic leukemia; CB, cord blood; CIK, cytokine-induced killer cells; CR, complete response; DC, dendritic cell; GFCH, Guangzhou Fuda Cancer Hospital, China; HCC, hepatocellular carcinoma; HUB, Humboldt-Universität zu Berlin, Germany; ITT, Intention-to-treat analysis; MM, multiple myeloma; NSCLC, non-small-cell lung carcinoma; OS, overall survival; PD, partial disease; PFS, progression-free survival; RCC, renal cell carcinoma; RFS, recurrence-free survival; SD, stable disease; SUSM, Sungkyunkwan University School of Medicine, Seoul, Korea; TMUC, Tianjin Medical University Cancer Institute and Hospital, Tianjin, China*.

Various CIK clinical trials have combined CIK cells with other therapies, including mAb therapies, chemotherapies, radiotherapy, and dendritic cell (DC) therapy. An in-depth review of all CIK clinical studies is beyond the scope of this article because more than 100 clinical trials of CIK have been reported, and readers are referred to Gao et al (85), for a review (91). Some examples of key clinical studies are listed in Table 4. In 2012, a randomized phase II clinical study showed that CIK therapy could enhance the efficacy of conventional chemotherapy in patients with NSCLC (87). The study was designed to evaluate the clinical efficacy of CIK cell immunotherapy following regular chemotherapy in patients with NSCLC after surgery. Among early-stage patients, the 3-year OS rate and median OS time in the immunotherapy arm were significantly higher than those in the no immunotherapy arm. Among the advanced-stage patients, the 3-year OS rates of immunotherapy arm were significantly higher than those of the no immunotherapy arm. Lee et al. demonstrated that adjuvant immunotherapy with anti-CD3-activated CIK cells could increase the recurrence-free survival (RFS) and OS of patients with HCC (88). Patients who received CIK immunotherapy after curative treatment for HCC had a 14-month median RFS benefit compared to the no immunotherapy control group. Another recent report described results of a study comparing chemotherapy followed by CIK immunotherapy including DCs versus chemotherapy alone in stage IV breast adenocarcinoma (89). Over a 10-year period, a total of 368 patients meeting the inclusion criteria were assigned to the study groups. CIK immunotherapy consisted of monocyte-derived autologous DC plus freeze-thawed tumor lysate, with CIK prepared from non-adherent cells from the DC culture process. OS rates were significantly prolonged in patients in the DC-CIK treatment group compared with the patients in the control chemotherapy alone group. Finally, a recent phase III trial has evaluated CIK immunotherapy with radiotherapy-temozolomide (TMZ) versus TMZ alone for the treatment of newly diagnosed glioblastomas (90). A total of 180 patients were randomly assigned to the CIK immunotherapy (*n* = 91) or control group (*n* = 89). The addition of CIK cell immunotherapy to standard chemoradiotherapy with TMZ resulted in a statistically significant improvement in PFS. However, the CIK immunotherapy group did not show evidence of a beneficial effect on OS rates. Other notable studies in clinical development but yet to report clinical results are seeking to combine CIK with mAb anti-PD1/PDL1 strategies (NCT02886897, NCT03190811, NCT03360630, NCT03282435, and NCT03146637).

Studies have begun to explore the use of CIK cells as a CAR cell carrier. Multiple preclinical studies have demonstrated promising antitumor activity against a variety of tumors and targets [for example, Ref. (92–96)]. Formula Pharmaceuticals recently announced the opening of the first clinical trial of CAR-modified allogeneic CIK cells using its non-viral gene delivery approach (http://www.formulapharma.com/news/2017_09_18_formula_press_release.asp).

The next few years will see clinical results from CAR-modified NK cell and CAR-modified CIK cell trials and will determine if these approaches are efficacious and can complement or perhaps replace CAR-T/TCR-T approaches.

### Gamma Delta T Cells

Gamma-delta (γδ) T cells represent less than 5% of circulating T cells and unlike conventional αβ T cells, the TCR repertoire of γδ T cells is very restricted. They can be cytotoxic and secrete cytokines like conventional αβ T cells, but γδ T cells do not require MHC antigen presentation for antigen recognition. γδ T cells are also more widely distributed in tissues throughout the body, in addition to also being present in the typical T cell compartments, the lymph nodes, and spleen, where most αβ T-cells reside. Thus, γδ T cells could potentially offer differentiated activity from αβ T cells, and some investigators are beginning to explore their utility as CAR carriers. Indeed, one specific subset of γδ T cells identified by their expression of a Vg9Vd2 TCR can be expanded using bisphosphonates and are known to recognize in an MHC-independent manner phosphoantigens, and these tend to be enriched in tumors (97). The MHC-independent nature of the Vg9Vd2 antigen recognition also means that they are not alloreactive and therefore should not cause GvHD. Deniger et al. recently reported on a methodology to expand polyclonal γδ T cells *in vitro* and electroporation with Sleeping Beauty transposon and transposase to enforce expression of a CD19 CAR (98). The demonstrated killing of CD19^+^ tumor cell lines and adoptive transfer of CAR γδ T cells reduced growth of CD19^+^ leukemia xenografts in mice. Clinical translation of CAR γδ T cell approaches will be needed to determine if these cell carriers provide advantages over CAR αβ T cells. In that regard, Chen and colleagues at the Fuda Cancer Hospital, Guangzhou, China have recently opened four studies for enrollment to assess safety and efficiency of autologous peripheral blood mononuclear cell-derived γδ T cells against lung cancer (NCT03183232) and liver cancer (NCT03183219), breast cancer (NCT03183206), and pancreatic cancer (NCT03180437) respectively (Table 5).

**Table 5:** Summary of some key clinical studies evaluating gamma-delta T cell therapies.

Center	Clinicaltrials.gov identifier	Product	Indication	Outcome
Fuda Cancer Hospital	Phase I, NCT03183232	Autol. PBMC-derived γδ T cells	Lung cancer	Study open to enrollment
Fuda Cancer Hospital	Phase I, NCT03183219	Autol. PBMC-derived γδ T cells	Liver cancer	Study open to enrollment
Fuda Cancer Hospital	Phase I, NCT03183206	Autol. PBMC-derived γδ T cells	Breast cancer	Study open to enrollment
Fuda Cancer Hospital	Phase I, NCT03180437	Autol. PBMC-derived γδ T cells	Pancreatic cancer	Study open to enrollment

*Autol., autologous; PBMC, peripheral blood mononuclear cells; γδ, gamma–delta*.

## Cell and Gene Therapies Using Genome Engineering

A further step in simplifying supply and broadening access to donor cells is to engineer partially matched allogeneic cells. This approach is attractive for situations where dramatic clinical efficacy can be mediated rapidly and where perhaps sustained persistence of the cell product is not a requirement. One such example was recently reported whereby unmatched allogeneic donor T cells were lentivirally transduced to express an anti-CD19 CAR, followed by gene editing with TALENs to remove expression of the endogenous TCR alpha chain to prevent GvHD together with removal of CD52 to render them insensitive to the lymphodepleting agent Alemtuzumab (99). These TCR-ablated T cells were then administered to two relapsed refractory B-acute lymphoblastic leukemia patients as temporary “bridge to transplant” designed to eradicate their leukemia before a subsequent planned allogeneic HSCT. Recently, it has also been shown that gene editing could be used to generate high numbers of redirected NY-ESO-1 T cells with a predominant stem and memory T cell phenotype that were tested preclinically *in vitro* and in a mouse model of MM without inducing GvHD (100).

The “holy grail” would be an entirely “universal donor,” long-lived cell product providing an “off-the-shelf” cell therapy acceptable to any patient irrespective of their HLA and TCR specificity. Technically, genome editing technology offers the possibility to engineer such universal donor cells in a controlled manner. Cells could be modified *ex vivo* using gene editing to knockout expression of HLA class I and potentially HLA class II molecules to prevent GvHD that could otherwise lead to graft rejection. It has been shown that elimination of HLA class I molecules using zinc finger nucleases was possible in primary T cells as well as in embryonic stem cells (101, 102). Furthermore, the removal of HLA-A molecules in HSC had no impact on their function as the cells could engraft in immuno-compromised mice (103). Depending upon the donor cell type, the removal of other molecules such as the TCR that could induce GvHD would be required. Additionally, other cell engineering may be necessary such as the introduction of a β-2 microglobulin (β-2M) gene fusion to a non-polymorphic HLA-E, F, or G gene in order to avoid NK cell-mediated lysis of the cell product that would otherwise occur if the engineered cells are completely devoid of class I molecules (102, 104).

Zhao, June and colleagues have reported on preclinical studies combining lentiviral delivery of a CAR with electroporation of Cas9 mRNA and gRNAs targeting endogenous TCR, β-2M, and PD1 simultaneously, to generate gene-disrupted allogeneic CAR T cells deficient of TCR, HLA class I molecule, and PD1 by multiplex genome editing (105, 106). A clinical trial is planned to be undertaken at the University of Pennsylvania, University of California San Francisco, and the MD Anderson Cancer Center, USA. Lu and colleagues at Sichuan University’s West China Hospital in Chengdu in China have reported the first treatment with CRISPR-modified autologous peripheral blood T cells in lung cancer patients that had been disrupted for PD1 using CRISPR/Cas9 (NCT02793856). These and other early studies will evaluate the safety of genome-edited T cells and pave the way for the more extensive multiplex genome editing required to create universal cell products.

Once clinically validated, the genome editing solutions could equally be applied to different cell types such as T cells, DCs, or HSC. However, it remains to be demonstrated that such “universal” cells could readily engraft and provide long-term protection without inducing toxicity. In addition, the less than 100% efficiency achieved for each edit will require purification of “fully edited” from “partially edited” cells, which is a significant obstacle that is yet to be resolved. There may also be unidentified molecules that may need to be edited for a cell to be truly “invisible” to the host immune system and persist for the life of the individual.

## Cell Therapy to Control Infection

Infections are still one of the main complications post-HSCT contributing to mortality and morbidity of the procedure. Therefore, a lot of efforts have been dedicated in developing cell therapy approaches to control infection such as DLI as an alternative to more conventional therapies. Notably, it has been shown by several groups that the infusion of matched donor-derived virus-specific T cells specific for cytomegalovirus, Epstein–Barr virus, or adenovirus can restore virus-specific immunity and control infection or could be used prophylactically in transplanted patients (107–114) (Table 6). In addition, the use of multivirus-specific T cells has also been reported to be effective in either preventing or controlling viral infection (115–118) (Table 6). However, this approach is not applicable to seronegative donors, patients undergoing immunosuppressive regimens, or for urgent use because of the time required to generate the cell product. In these specific conditions, the use of partially matched allogeneic virus-specific T cells has shown great promise and efficacy in controlling infection (116, 119, 120) (Table 6), highlighting the possibility to develop banks of allogenic virus-specific T cells (113, 121) whereby the virus-specific T cells of one donor could be expanded and banked for immediate use, which could allow treating several patients with the cells from the same donor thus decreasing the cost of the therapy.

**Table 6:** Summary of some key published clinical studies evaluating virus-specific T cell therapies.

Reference	Center	Clinicaltrials.gov identifier	Product	Indication	Outcome	Toxicity
(107)	UCL	Phase I, NCT01115816	CMV-specific T cell lines	Preemptive treatment of CMV disease post-HSCT (*n* = 16)	8/16 cleared without anti-virals, 8/16 clears with ganciclovir, *in vivo* expansion, viral immunity restored	
(108)	Tübingen University	Phase I	Virus-specific donor T cells	Treatment of adenovirus infection post-HSCT (*n* = 9)	Successful in 5/6 patients with durable clearance/decrease of viral copies	1 patient developed grade II skin GvHD
(109)	UCL	Phase III, NCT01077908	CMV-specific T cell lines or CMV-specific selected T cells	CMV infection, prophylactic therapy (*n* = 91)	*In vivo* expansion, decreased viral titers, and low incidence of CMV reactivation	
(111)	Munich University	Phase I	EBV-specific T cells	Treatment of PTLD (*n* = 6)	3/6 CR	
(115),	Shanghai University	Phase I	CMV/EBV-specific immune effector cells	Preemptive treatment of CMV or EBV disease (*n* = 3)	All 3 patients were CMV/EBV free for up to 18 months	
(116)	UCL	Phase I	Third party virus-specific T cells	Treatment of adenovirus infection (*n* = 1)	Effective response	Induction of GvHD
(114)	Wurzburg University	Phase I, EudraCT-No. 2006-006146-34	Donor or third party CMV-specific selected T cells	Treatment of CMV infection post-HSCT (*n* = 16)	65 and 25% CR or PR rate observed, respectively	
(117)	Munich, Hannover, Regensburg, Wurzburg University	Phase I	Tri-virus-specific T cells	Treatment of CMV, EBV, or adenovirus infection post-HSCT (*n* = 10)	80% CR rate observed	
(118)	Baylor College	Phase I	Multivirus-specific T cells	Treatment of CMV, EBV, BK, HHV6, or adenovirus infection post-HSCT	94% clinical response rate	
(119)	Baylor College	Phase I	Third party virus-specific T cell lines	Treatment of CMV, EBV, or adenovirus infection post-HSCT (*n* = 11)	74% CR or PR rate at 6 weeks postinfusion	

*CMV, cytomegalovirus; CR, complete response; EBV, Epstein–Barr virus; GvHD, graft versus host disease; HHV6, human herpes virus 6; HSCT, hematopoietic stem cell transplantation; PR, partial response; PTLD, post-transplant lymphoproliferative disorder; UCL, University College London*.

## Cell and Gene Therapies to Induce Tolerance

Several cell types are capable to regulate immune responses and thus induce tolerance. NK cells have shown to have regulatory functions aside their cytotoxic activity in the context of HSCT, impacting on acute GvHD (60). In addition, different studies suggest that a higher number of NK cells and NK cell alloreactivity might reduce GvHD development (122–124). Moreover, double negative (DN) T cells have regulatory functions and play key roles in tolerance induction post-transplantation and in autoimmune diseases (125, 126). DN T cells have been reported to suppress immune responses, in particular T cell responses and can improve allograft survival in experimental mouse models of cardiac transplantation (127). Mesenchymal stem cells (MSC) are multipotent stem cells that also have anti-inflammatory and regenerative properties. Their regulatory functions have been evaluated using *in vitro* and *in vivo* disease models (128). In addition, the infusion of autologous and allogeneic MSCs has been tested in numerous phase I studies as immunosuppressive regimen in the context of organ transplant as well as treatment for GvHD and different autoimmune diseases (129, 130).

Regulatory T cells are key players in inducing tolerance and maintaining immune homeostasis. They constitute 5–10% of CD4 T cells in peripheral blood and are defined as CD4^+^ CD25^+^ CD127lowFoxP3^+^ (131). They have been shown to be able to regulate the functions of numerous immune cells such as CD4/CD8 T cells, DCs, NK cells, and B cells (132). Consequently, they have been evaluated when considering a cell therapy approach to induce tolerance especially for autoimmune diseases. Treg therapies have been shown to be safe and feasible with some promising results in clinical trials using autologous Tregs for GvHD (133–136) and autoimmunity such as diabetes (137, 138) and Crohn’s disease (139) as well as third-party allogeneic Tregs (136) (Table 7). All clinical studies have been done using polyclonal expanded Tregs; however, it has been shown that antigen-specific Tregs are more potent at suppressing undesired immune responses as compared to polyclonal Tregs. Some groups have studied the possibility to generate antigen-specific T cells by transferring a CAR into polyclonal expanded Tregs and showed their potency *in vitro* as well as in animal models (140–143). However, this approach is still to be tested clinically.

**Table 7:** Clinical studies evaluating Tregs therapies.

Reference	Center	Clinicaltrials.gov identifier	Product	Indication	Outcome	Toxicity
(133)	University of Gdansk	Phase I	Expanded donor polyclonal Tregs	GvHD (*n* = 2)	Safe, decreased use of immunosuppression for cGvHD, temporary impact on aGvHD	
(144)	University of Minnesota	Phase I, NCT00602693	Expanded third-party polyclonal UCB Tregs	GvHD (*n* = 23)	Reduced incidence of grade II–IV aGvHD (43 versus 61%)	Increased incidence of infection
(136)	University of Minnesota	Phase I, NCT00602693	Expanded third-party polyclonal UCB Tregs	GvHD (*n* = 11)	Reduced incidence of grade II–IV aGvHD (9 versus 45%)	
(134)	University of Perugia	Phase I, Protocol No 01/08	Donor polyclonal Tregs	GvHD (*n* = 28)	Safe, GvHD prevention in the absence of immunosuppression, improved reconstitution	2/26 developed > grade II aGvHD
(135)	Milan, TIGET	Phase I, ALT-TEN trial, registration number IS/11/6172/8309/8391	Donor Il-10 anergized T cells [peripheral T regulatory type 1 (Tr1) cells]	Fast immune reconstitution in 5 patients, safe	Transient GvHD in immune-reconstituted patients	
(137)	University of Gdansk Phase I	Phase I, ISRCTN06128462	Expanded autologous polyclonal Tregs	Type I diabetes (*n* = 12)	Safe, 66% of patients remained in remission during the follow-up	
(138)	San Francisco	Phase I, NCT01210664	Expanded autologous polyclonal Tregs	Type I diabetes (*n* = 14)	C-peptide levels persisted out to 2+ years after transfer in several individuals, long-term persistence of Tregs	
(139)	Lille University	Phase I/IIa, Eudract no. 2006-004712-44, Crohn’s and Treg Cells Study [CATS1]	Ova-specific expanded autologous Tr1	Crohn’s disease (*n* = 20)	Response in 40% (8/20) of treated patients	
(145)	San Francisco	Phase I, NCT02088931	Expanded autologous polyclonal Tregs	Kidney transplantation (*n* = 3)	Safe and well tolerated	

*aGvHD, acute graft versus host disease; cGvHD, chronic graft versus host disease; Ova, ovalbumin; Tr1 cells, type 1 regulatory cells; Tregs, regulatory T cells*.

Another approach to induce tolerance could be the transfer of a chimeric autoantibody receptor (CAAR) into T cells to induce cytotoxicity against autoreactive T or B cells. Such an approach has demonstrated exciting results *in vitro* and *in vivo* in the case of CAAR T cells redirected against desmogelin-3 (DSG3) as a therapeutic approach for pemphigus vulgaris where B cells secrete autoantibodies against DSG3, which is an antigen expressed in the skin and mucosa (146). This study showed effective killing of autoreactive cells by DSG3-CAAR T cells *in vitro* and *in vivo* while sparing keratinocytes with no off-target effects. This approach showed promising results to potentially treat autoantibody mediated immune diseases.

Numerous cell types with regulatory functions have now been tested in preclinical studies showing promising results. Further evaluation in clinical studies is warranted but these data will be key to evaluate whether gene engineering cell therapeutics could be as successful in inducing tolerance in chronic diseases as these types of approaches have been for rare diseases and oncology.

## Concluding Remarks

After many years of endeavor, the field of cell and gene therapy is starting to deliver remarkable clinical benefits in some patients, particularly in certain hematological malignancies. While this is a very exciting moment for the field, more work remains to be done to further develop and optimize therapies. More relevant disease models that can help predict not only efficacy but also adverse effects are much needed. In addition, making gene engineering cell therapeutics safer is key by adding, for example, safety or kill switch to the therapy or expression control elements. As this review shows, many cell types are being explored in early clinical trials and the results of these studies will provide important insights. A major factor in realizing the potential of cell and gene therapies in large numbers of patients and in more common diseases is the efficient and cost-effective manufacturing of gene engineering cell therapeutics. Advances in automation are a key component that needs to be addressed (147). The pace of clinical development is accelerating and this means that the next few years will provide many important learnings that will hopefully translate into new therapeutic options for patients.

## Author Contributions

AS, LJ, and TC all contributed to write the manuscript.

## Conflict of Interest Statement

All authors are employee of GlaxoSmithKline.
